# Production of Prophylactic Nanoformulation for Dental Caries and Investigation of Its Effectiveness by In Vitro and In Silico Methods

**DOI:** 10.3390/pharmaceutics17020167

**Published:** 2025-01-27

**Authors:** Yasemin Budama-Kilinc, Ozan Baris Kurtur, Bahar Gok, Serda Kecel-Gunduz, Sengul Alpay-Karaoglu, Pınar Yılmaz Atalı, Murat Kartal

**Affiliations:** 1 Department of Bioengineering, Faculty of Chemistry and Metallurgy, Yildiz Technical University, 34220 Istanbul, Turkey; bahar.991@hotmail.com; 2 Health Biotechnology Joint Research and Application Center of Excellence, 34220 Istanbul, Turkey; pinar.atali@marmara.edu.tr; 3 Department of Bioengineering, Graduate School of Natural and Applied Science, Yildiz Technical University, 34220 Istanbul, Turkey; obkurtur@hotmail.com; 4 Physics Department, Faculty of Science, Istanbul University, 34134 Istanbul, Turkey; skecel@istanbul.edu.tr; 5 Biology Department, Recep Tayyip Erdogan University, 53100 Rize, Turkey; sengul.karaoglu@erdogan.edu.tr; 6 Department of Restorative Dentistry, Faculty of Dentistry, Marmara University, 34854 Istanbul, Turkey; 7 Faculty of Pharmacy, Bezmialem Vakif University, 34093 Istanbul, Turkey; mkartal@bezmialem.edu.tr

**Keywords:** essential oil, dental caries, controlled release nanoformulation

## Abstract

**Background/Objectives:** This study aimed to develop cinnamon bark essential oil (CEO), orange peel essential oil(OEO) and the combination of these two essential oils (OEO-CEO) loaded PLGA nanoparticles to prevent dental caries and to investigate their effectiveness in silico and in vitro methods. **Methods:** EO loaded PLGA nanoparticles were produced by single emulsion method. Detailed characterization studies were performed using different methods, and the controlled release profile was obtained. The antibacterial activity of the developed formulations was investigated on *S. mutans* and *L. casei* strains by in vitro and in silico methods. Additionally, the interaction mechanisms of EOs with DNA were evaluated. **Results:** Our findings showed that the average droplet size of EO-loaded PLGA nanoparticles varied between 243.1 ± 0.60 nm and 219 ± 4.49 nm, while PdI values varied between 0.069 ± 0.039 and 0.032 ± 0.01. In addition, the developed nanoparticles had high encapsulation efficiency (85.14% to 66.28%) and released the active ingredient in a continuous and controlled manner. Ames test showed that the genotoxicity of EOs was eliminated due to the encapsulation of EOs in PLGA nanoparticles and antibacterial tests showed that OEO-CEO-loaded PLGA nanoparticles were effective on L. casei and S. mutans. The antibacterial activity of EOs was also supported by in silico studies. Finally, it was revealed that EOs showed potential as antibacterial agents by interacting with DNA. **Conclusions:** The results showed that OEO-CEO-loaded PLGA nanoparticles have the potential to be a suitable nanoformulation for developing mouthwash or toothpaste for the prevention and treatment of dental caries.

## 1. Introduction

Dental caries is a chronic microbial illness affecting both children and adults worldwide and is one of the major oral diseases [1,2,3]. The tooth surface becomes colonized (biofilm) by the bacteria that cause dental caries. The complex tooth structure is harmed by the presence of fermentable carbohydrates like fructose and sucrose [4,5]. *Streptococcus* and *Lactobacillus* bacteria not only cause tooth decay but also play an important role in the progression of tooth decay [6]. The most prevalent bacteria in saliva and tooth plaque, *Streptococcus mutans*, is connected to initial caries [7]. *Lactobacillus* species comprise 1% of the oral microbiota [8]. *Lactobacillus casei* is essential because of its caries-forming properties. *Lactobacillus* is acidogenic, and unlike their cariogenic partner, *S. mutans*, they alone do not adhere to the tooth surface efficiently. However, with the presence of *S. mutans* and the additional primary colonizers, their capacity to establish on tooth surfaces can be substantially augmented, though variations exist among different species. Therefore, they are more common in advanced caries lesions [9].

There is a global need for safe, effective, and economical alternative preventive treatment options due to the rising incidence of oral diseases, particularly in developing countries, the resistance of pathogenic bacteria to currently used antibiotics and chemotherapeutics, and opportunistic infections in patients with compromised immune systems [5,10]. Despite the few commercially available therapeutic agents, these substances can change the oral microbiota and have unfavorable side effects such as vomiting, diarrhea, and tooth discoloration [11]. Moreover the development of drug resistance against currently used antibiotics by various bacterial species that cause oral diseases is seen as a significant risk [12]. For example, it was determined that *S. mutans* strains in samples taken from fifty patients with dental caries showed high resistance to amoxicillin, ampicillin, erythromycin, tetracycline, and penicillin, and these strains showed 80% resistance to more than three antimicrobial agents [13]. Furthermore, a cross-sectional study on bacteria isolated from dental caries reported that *S. mutans*, *Streptococcus epidermidis*, *Streptococcus oralis* and *Lactobacillus acidophilus* strains showed high resistance to penicillin by 82.2%, tetracycline by 88.4%, imperium by 100% and penicillin by 82.5%, respectively [14]. For this reason, new approaches continue to search for alternative products to prevent dental caries [15].

DNA is the pharmacological target of many drugs currently in use and used in clinical trials [16]. This is because DNA has two main functions in the cell: replication and transcription. Replication and transcription are vital for cell survival, reproduction, and the proper functioning of the entire body. Drugs that interact with DNA cause the inhibition of DNA. In other words, it inhibits replication or protein synthesis and leads to cell death. Therefore, DNA inhibition is essential for antitumor and antimicrobial agents [17]. In recent years, research on the identification of molecules interacting with DNA has received interest as a novel approach, and its application in industries, including biotechnology, nanotechnology, and the pharmaceutical sector, has been stressed [18].

For thousands of years, plants have been utilized as a traditional medicine for treating and preventing numerous ailments in many different parts of the world [19]. According to the World Health Organization (WHO), due to their accessibility, affordability, and lack of side effects, herbal medications are preferred by around 80% of the global population for the prevention of various diseases [20,21]. Plants are primarily used in dentistry to help prevent tooth decay and gum disorders due to their antibacterial effects [22,23]. In this respect, natural bioactive substances obtained from plants are currently used as additives. Among them, alkaloids, polyphenols, flavonoids, tannins, essential oils (EOs), terpenes, and terpenoids, as secondary metabolites, exhibit antimicrobial activity through different mechanisms of action [24]. For example, alkaloids, which are investigated as a natural antibiotic type, are nitrogen-containing heterocyclic compounds. They have significant advantages such as structural diversity, broad antibacterial spectrum, rare side effect profiles and low tendency to trigger drug resistance. Alkaloids show activity through mechanisms of action such as inhibition of bacterial cell wall synthesis, metabolism, nucleic acid and protein synthesis or alteration of cell membrane permeability [25]. Polyphenols with a wide structural diversity can show antimicrobial activity at the cellular level by damaging the cell wall, disrupting the cell membrane structure and function, inducing oxidative stress, inhibiting macromolecule synthesis, disrupting energy metabolism and triggering apoptosis-like death mechanisms. Moreover, polyphenols are known to show antibiofilm properties by affecting the c-di-GMP signaling system or quorum sensing system [26]. On the other hand, EOs derived from plants have drawn more attention recently [27].

EOs are one of the plant extracts used for centuries to prevent various medical and dental issues [27,28,29]. They are known for their bactericidal, sedative, virucidal, anti-inflammatory, fungicidal, spasmolytic, local anesthetic, and analgesic properties. The presence of complex chemical structures consisting of several groups, such as terpenoids and terpenes, aromatic and aliphatic components, all characterized by low molecular weight, may explain their successful bacteriostatic and bactericidal effects [30]. Interestingly, some of the research has proven that EOs can be beneficial against multidrug-resistant bacteria [31,32]. Among the plants investigated for the antimicrobial effects of EOs are cinnamon bark EO and orange EO. It is suggested in the literature that the components in cinnamon EO and cinnamon extracts may be beneficial in preventing caries and periodontal diseases with their antimicrobial activity against oral pathogens. Furthermore, it is known that cinnamon EO and cinnamon extracts contain cinnamaldehyde as the major component, and this compound has antibacterial properties. Therefore, more research should focus on the clinical use of oral care products containing cinnamon EO [33]. Moreover, it has been determined that EOs obtained from orange peel have a strong antimicrobial effect on various pathogens. This effect is thought to be due to D-limonene, the main component of the EO [34]. In addition, it was stated that these EOs obtained from orange peel have antimicrobial and antimycotic properties, which have a significant potential for use in mouthwash [35,36].

Despite the antibacterial activities of EOs, the hydrophobicity, volatility, toxicity and pungent odor of EOs limit their direct use [37,38]. Nanoparticle dosage forms, which can solve the stability problem of EOs, mask their pungent odor, and contribute to increasing bioavailability, can overcome this situation [39]. Nanoparticles have attracted great interest due to solving the toxicity problem of active ingredients, drug targeting efficiency, increased bioavailability, and biodistribution [40]. In addition, metal, metal oxide, calcium fluoride, chitosan and PLGA (poly-lactic-co-glycolic acid) polymeric nanoparticles are actively used as antimicrobial additives in dentistry. These nanoparticles perform effective interactions with bacterial cells due to their high surface area-to-volume ratio and high charge density [24]. Here, PLGA has received Food and Drug Administration (FDA) approval for its biodegradability and biocompatibility. It is the best-defined biomaterial available for drug delivery in terms of design and performance [41].

In our study, formulations in PLGA nanoparticle dosage form were synthesized by using cinnamon bark EO (CEO), orange peel EO (OEO), and the combined state of these two EOs (OEO-CEO). Characterization studies of the developed PLGA nanoparticles were carried out with various spectroscopic (UV–Vis spectroscopy, dynamic light scattering spectroscopy (DLS)) and transmission electron microscope (TEM) imaging methods. The antibacterial activity of the developed formulations was investigated on *S. mutans* and *L. casei* strains by in vitro and in silico methods. In addition to antibacterial studies, the interaction of EOs with DNA was also evaluated since the interaction of antibacterial agents with DNA is essential for antibacterial activity.

## 2. Materials and Methods

### 2.1. Materials

Both essential oils were supplied from Fitovizyon Doğal ve Sağlıklı Yaşam San. Tic. Ltd. Şti. (FitoBio- https://www.fitobio.com/ access on 14 January 2025) (Istanbul, Türkiye). The quality of essential oils complies with ISO and European Pharmacopoeia quality monographs. Resomer^®^ RG 504 H type PLGA with acid termination, 50:50 lactide:glycolide ratio, 38,000–54,000 molecular weight and 0.45–0.60 dL/g inherent viscosity was purchased from Sigma-Aldrich (Product Number: 719900) (St. Louis, MO, USA). Polyvinyl alcohol (PVA), dichloromethane (DCM), calf thymus DNA (CT-DNA) and ethidium bromide, sodium ammonium phosphate tetrahydrate, magnesium chloride hexahydrate, potassium phosphate, disodium hydrogen phosphate dihydrate, 4-nitro-o-phenylenediamine, L-histidine, D-biotin, sodium dihydrogen phosphate monohydrate, sulfate heptahydrate, citric acid monohydrate and sodium azide were purchased from Sigma Aldrich (St. Louis, MO, USA). Tris base, Ethylenediaminetetraacetic acid (EDTA), and sodium chloride (NaCl) were purchased from Merck Millipore (Darmstadt, Germany). pBR322 Plasmid DNA was purchased from Thermo Fisher (Waltham, MA, USA). Agar was purchased from Difco and Nutrient broth was purchased from Oxoid (Basingstoke, UK). *S. typhimurium* TA100 and *S. typhimurium* TA98 mutant strains were purchased from Moltox (Boone, NC, USA).

### 2.2. Methods

#### 2.2.1. Standardization and Characterization of CEOs and OEOs

Standardization and characterization studies of EOs were carried out at Bezmialem University Phytotherapy Center under the supervision of Prof. Dr. Murat Kartal. Gas chromatography mass spectrometry (GC-MS) was used to identify EO components, and gas chromatography flame ionization detector (GC-FID) was used to determine relative percentages. A 5% solution of each EO was prepared in hexane, and the analyses were completed [42]. The analysis conditions and detector characteristics are stated in Table S1.

#### 2.2.2. Determining the Standard Curve of EOs

Standard curves of EOs were determined by using a UV–Vis spectrophotometer (Shimadzu, Kyoto, Japan). Here, different concentrations of EO solutions were prepared in methanol. For OEO, seven different concentrations (0.9765625, 1.953125, 3.90625, 7.8125, 15.625, 31.25, and 62.5 µg/mL) were prepared, and the absorbance value of all concentrations was measured at 207 nm (n = 3). For CEO, eight different concentrations (0.0390625, 0.078125, 0.15625, 0.3125, 0.625, 1.25, 2.5, and 5 µg/mL) were prepared, and the absorbance value of all concentrations was measured at 285 nm (n = 3). The obtained absorbance values were used to determine the standard curves for both EOs. The equation of the standard curve was used in encapsulation efficiency, loading capacity and in vitro release studies [38].

#### 2.2.3. Fabrication of EO-Loaded PLGA Nanoparticles

The single emulsion (*o*/*w*) method was used to develop three different EO-loaded PLGA nanoparticles (OEO-loaded PLGA, CEO-loaded PLGA, and OEO-CEO-loaded PLGA nanoparticles) [43]. A total of 250 mg of PLGA was dissolved in 10 mL of DCM, and then 50 mg of EO was added to 2 mL of PLGA solution and mixed on a magnetic stirrer. The EO-PLGA mixture was then slowly added to 6 mL of PVA (3%) solution with the help of an injector. After this process, the mixture was homogenized by ultrasonication for 5 min by applying 70 W energy. The solvent was then removed by stirring in a magnetic stirrer at 250 rpm for 16 h. The nanoparticles were centrifuged three times at 8000 rpm at 4 °C for 40 min. Nanoparticles were freeze-dried in a lyophilizer for 48 h at −60 °C for further analysis.

#### 2.2.4. DLS Analysis of EO-Loaded PLGA Nanoparticles

In our study, the DLS method was used to determine the average particle size, polydispersity index (PdI), and zeta potential of blank PLGA, OEO-loaded PLGA, CEO-loaded PLGA, and OEO-CEO-loaded PLGA nanoparticles. DLS analysis was performed using a Zeta Sizer Nano ZS (Malvern Instruments, Worcestershire, UK) instrument equipped with a 4.0 mV He-Ne laser operating at 25 °C. In order to determine the average particle size and PdI values of nanoparticles, 1 mL of sample was added to the DTS0012 cuvette (Malvern Instruments, Worcestershire, UK); for the zeta potential values, measurements were made by adding 0.75 mL of sample to the DTS1060 cuvette (Malvern Instruments, Worcestershire, UK).

#### 2.2.5. TEM Analysis

Morphological analysis of OEO-loaded PLGA, CEO-loaded PLGA, and CEO-OEO-loaded PLGA nanoparticles was performed using TEM (JEOL JEM-1400 Plus) (Tokyo, Japan). A total of 3 mg of dry sample was dispersed in isopropyl alcohol. A total of 1 µL of the dispersed sample was dropped onto a carbon film-supported copper grid and analyzed after evaporating the solvent. TEM images were taken by operating at 80 kV voltage.

#### 2.2.6. Stability Test

EOs-loaded PLGA nanoformulations were frozen in a lyophilizer at −60 °C for 2 h to determine physicochemical stability and dried for 48 h without a cryoprotectant agent. Then, dry nanoformulations were stored at 5 ± 3 °C, 25 ± 2 °C 60% RH, 40 ± 2 °C 75% RH, and DLS analysis was performed on the first day, first month, second month, and third month time periods. Changes in average particle size, PdI, and zeta potential values compared to the initial states of the formulations were used to monitor stability.

#### 2.2.7. Determination of Encapsulation Efficiency and Loading Capacity of EO-Loaded PLGA Nanoparticles

The encapsulation efficiency of the synthesized nanoparticles was achieved using the solvent extraction method [44]. Briefly, EO-loaded PLGA nanoparticles were dissolved in methanol, vortexed, and centrifuged at 8000 rpm for 15 min. Then, the supernatants were collected, the UV–Vis spectrometer was measured, and absorbance values were determined. These absorbance values were used to calculate the amounts of EOs in the supernatant through the standard curve equations of OEO and CEO. Blank nanoparticles were used as references. Encapsulation efficiency was calculated using Equation (1); loading capacity was calculated using Equation (2).

Encapsulation Efficiency (%) = (Actual EOs Loaded)/(Theoretical EOs Loaded) × 100,(1)

Loading Capacity (%) = (EOs Mass in Particles)/(Mass of Particles) × 100,(2)

#### 2.2.8. In Vitro Release Profile of EO-Loaded PLGA Nanoparticles

The dialysis membrane method was used to determine the in vitro release profile of EO-loaded PLGA nanoparticles [38,45,46]. Each EO-loaded PLGA nanoparticle (3 mg) was dispersed in distilled water (1 mL) and placed in dialysis capsules. The release was carried out in phosphate buffer (PBS) at pH = 7.4 and, since the samples were EOs, at room temperature [47] in a horizontal shaking water bath at 120 rpm. Samples taken from the release medium at the specified time intervals were analyzed using a UV–Vis spectrometer, and the percent release values were calculated using Equation (3).

Release (%) = (Amount of released EO)/(Amount of total EO) × 100,(3)

#### 2.2.9. Ames Test

In our study, TA98 and TA100 mutant strains of *S. typhimurium* were used to evaluate the genotoxicity of EOs and EO-loaded PLGA nanoparticles. Concentrations of 0.125, 0.250, 0.5 and 1 mg/mL were used to evaluate the genotoxicity of EO-loaded PLGA nanoparticles. EO concentrations loaded into the nanoparticles (0.10, 0.20, 0.40, and 0.80 mg/mL, respectively) were used to evaluate the genotoxicity of CEO and OEO. The genotoxicity of OEO-CEO was evaluated at 0.10, 0.20, 0.40, and 0.80 mg/mL concentrations, with a 1:1 ratio of OEO and CEO concentrations. In the study, ethanol for EO and distilled water for nanoparticles were used as the negative control. For positive controls, 4-nitro-*o*-phenylenediamine for TA98 and sodium azide for TA100 were used. The genotoxic potential of EOs and EO-loaded PLGA nanoparticles was evaluated by comparing the number of returned colonies on EO and EO-loaded PLGA nanoparticle plates with the number of returned colonies on negative control plates. Moreover, these results were evaluated statistically at the *p* ˂ 0.05 level with the Tukey test in the SPSS 22 program for Windows, according to Mortelman and Zeiger [48].

#### 2.2.10. Determination of Minimum Inhibitory Concentration (MIC)

In this study, the antibacterial activities of EOs, EO-loaded PLGA nanoparticles, and blank PLGA nanoparticles were determined by applying the minimum inhibitory concentration (MIC) method. A single colony was taken from each bacterium for viability controls and transferred to M.R.S medium for *L. casei* RSKK591 (Refik Saydam Hıfzıssıhha Institute, Ankara, Turkey) and brain heart infusion medium for *S. mutans* RSKK07038 (Refik Saydam Hıfzıssıhha Institute, Ankara, Turkey). Received colonies were incubated at 37 °C for 24 h as microaerophilic (growth at the minimum oxygen concentration). Bacterial solutions were prepared from overnight cultures taken the next day at a density of 0.5 McFarland to be used in the experiment [49].

The MIC is the lowest concentration of antibacterial agent that completely inhibits the apparent growth of the test strain under in vitro conditions [50]. While the MIC method has rarely been used for many years, it has recently started taking place among routine tests. However, it is an important method for effective and optimal treatment [51]. Here, 100 μL of M.R.S medium (Merck) for *L. casei* and brain heart infusion medium (Conda) for *S. mutans* were transferred to a flat-bottomed 96-well microplate. Stock solutions of CEO, OEO, CEO-OEO, blank PLGA nanoparticles, CEO-loaded PLGA nanoparticles, OEO-loaded PLGA nanoparticles, and CEO-OEO-loaded PLGA nanoparticles were prepared. Then, these stock solutions were serially diluted to obtain solutions with different concentrations, and 100 μL of solution from each concentration was transferred to the medium. The prepared cultures were diluted to 1 × 10^6^ cfu/mL, and 10 μL of each was taken and added to the solutions in the wells. Control groups were prepared for all tests. The plates were incubated at 37 °C for 24 h [52]. Plates were scanned with an ELISA reader (595 nm) to determine the presence of viable bacteria. The lowest concentration at which growth was 100% inhibited by the *S. mutans* and *L. casei* strains used was recorded as the MIC values of the substances [53].

#### 2.2.11. DNA Binding

Agents that bind to and cut DNA are important for bacterial and cancer diseases [54]. Therefore, the DNA binding activity of CEO and OEO was determined in our study. At room temperature, a DNA binding study was performed using the UV–Vis absorption titration method in Tris-HCl/NaCl buffer (pH 7.2). The experiment was carried out by adding increasing amounts of calf thymus DNA (CT-DNA) concentration and adding a fixed amount of CEO and OEO concentrations. When measuring the absorbance, equal amounts of CT-DNA were added to both the solutions containing the active ingredients and the reference solution to eliminate the self-absorbance of the CT-DNA. The EO-DNA solutions were incubated for 5 min at room temperature, and the absorption spectra were recorded.

#### 2.2.12. DNA Cleavage

DNA cleavage activity of EO and EO-loaded PLGA nanoparticles was determined by agarose gel electrophoresis method using pBR322 plasmid DNA. DNA cleavage properties of EOs and nanoparticle dosage forms of EOs encapsulated with PLGA polymer were determined both in the presence (oxidative cleavage) and absence (hydrolytic cleavage) of oxidizing agent (H_2_O_2_). While H_2_O_2_ was added in the oxidative cleavage test, autoclaved water was added in the hydrolytic cleavage test. The experiment was carried out by adding pBR322 DNA (0.1 µg/µL), substances, and water/H_2_O_2_ in Tris-HCl buffer (10 M, pH: 7.2) and incubating at 37 °C for 3 h. After incubation, samples were run in TBE (Tris-Boric acid-EDTA, pH: 8.0) buffer at 60 V for 1 h. The bands were then visualized with UV light [55].

#### 2.2.13. In Silico Molecular Docking Studies

Cinnamaldehyde (3-phenyl-2-propenal phenol; C_9_H_8_O), a phenylpropanoid, is the most abundant in the CEO used in our study. Therefore, cinnamaldehyde was chosen for our molecular docking study. The three-dimensional molecular structure of the active ingredient cinnamaldehyde was obtained from PUBCHEM (PUBCHEM ID: 637511). It was optimized using appropriate package programs, and its spatial geometry was determined to be in the most stable, most efficient, and most active state. Although OEO, another EO used in our study, has different active ingredients, the D-limonene component is the major component among these ingredients. The three-dimensional structure of D-limonene (C_10_H_16_) was obtained from PUBCHEM (PUBCHEM ID: 440917). In order to obtain the most active and stable geometry, it has been optimized by selecting appropriate theory levels and programs. These two optimized active ingredients were used as active ligands for molecular docking analysis. The antibacterial properties of these active ingredients, that is, their inhibitory activities against *S. mutans* and *L. casei*, were analyzed and evaluated by the in silico molecular docking method. The Maestro 11.4 Glide module Maestro Version 12.5.139, MMshare Version 5.1.139, Release 2020-3, Platform of the Schrödinger program was used in molecular docking studies.

For molecular docking analysis, the three-dimensional structure of our active receptors, *S. mutans* and *L. casei*, was taken from the protein data bank. Many three-dimensional structures of bacteria have been detected in the protein data dank (ID: 3CZC, 3IHK, 4TQX, 6J9U, 6O1A, 4DFR, 1LLC, etc.). Among these structures, the most suitable crystal structures with the best resolution for bacteria were selected as possible receptor structures. Using the “receptor preparation wizard in the Maestro Version 12.5.139, MMshare Version 5.1.139, Release 2020-3” in the Schrödinger Glide module, all water and other molecular structures, such as ions, were removed from the structure, and polar hydrogen atoms were added to the receptor. The bond patterns were determined, the charges were determined by PROPKA at neutral pH, and the selected receptor was fixed with an RMSD value of 0.3 Å; the heavy atoms were approached, and the OPLS3 force field was selected. In addition, by using the OPLS force field with the “LigPrep” tool in the Maestro “Maestro Version 12.5.139, MMshare Version 5.1.139, Release 2020-3”, also included in the Glide module of this program, Cinnamaldehyde, the main active component of CEO, and D-limonene ligands, the main active component of OEO, were formed in 32 different conformers, that is, their spatial positions, at neutral pH. All these generated conformers were used for docking. The most active ligand–receptor binding areas were determined by creating a grid to determine the active binding areas of the receptors, that is, the most active binding areas where Cinnamaldehyde and D-limonene ligands would bind to *S. mutans* and *L. casei* and inhibit the bacteria. After the grid process, molecular docking analysis was applied to determine the binding affinities, binding sites, and binding mechanisms (by which type of bonds the binding occurs) of Cinnamaldehyde and D-limonene bindings to *S. mutans* and *L. casei*, and inhibition activities on *S. mutans* and *L. casei* were compared. By using the molecular docking analysis method, which is an in silico molecular modeling method, the antibacterial activities of cinnamon (*Cinnamomum cassia*) bark EO and orange (*Citrus sinensis*) EO were detected and revealed in three dimensions through atomic interactions. In this way, the different inhibition mechanisms of different EOs on different bacteria and their antibacterial activities were determined by simulation in a computer environment and compared with the experimental results obtained.

#### 2.2.14. Statistical Analysis

For genotoxicity tests, the results of the measurements (n = 3) obtained from the control and treatment groups were compared. Differences between group mean for the test system were evaluated with one-way ANOVA (analysis of variance) using the IBM SPSS Statistics 21 program. The significance level of the differences between the group mean was determined at the *p* < 0.05 level with the Tukey test according to the homogeneity of the variances. Also, mean values are presented as ±SD.

## 3. Results

### 3.1. OEO and CEO Compositions

The chemical components of OEO and CEO are shown in Table 1 and Table 2, respectively. A total of five compounds were detected in OEO, and their amounts were determined. GC-MS results revealed that the main phytochemical of OEO is limonene, which has a rate of 97.309%. Furthermore, a total of 18 compounds were detected in CEO and their amounts were determined. The main components of CEO are 70.267% cinnamaldehyde, 10.581% cinnamyl acetate, and 5.220% eugenol, respectively. The GC-MS chromatograms of both EOs are shown in Figure S1.

### 3.2. DLS Analysis Results

DLS results of blank PLGA, OEO-loaded PLGA, CEO-loaded PLGA and OEO-CEO-loaded PLGA nanoparticles are shown in Figures S2–S5. Blank PLGA nanoparticles have a average particle size of 212.6 ± 2.04 nm and a narrow particle size distribution with a PdI of 0.047 ± 0.031 (Figure S2a). The zeta potential value was −7.53 ± 0.35 mV (Figure S2b). The average particle size of the OEO-loaded PLGA nanoparticles was 243.1 ± 0.60 nm, the PdI value was 0.078 ± 0.06, and the zeta potential value was −6.02 ± 1.10 mV (Figure S3a,d). The average particle size of CEO-loaded PLGA nanoparticles was 235.9 ± 4.03 nm, the PdI value was 0.069 ± 0.039, and the zeta potential value was −6.10 ± 0.05 mV (Figure S4a,d). The average particle size of the OEO-CEO-loaded PLGA nanoparticles was 219 ± 4.49 nm, the PdI value was 0.032 ± 0.01, and the zeta potential value was −4.62 ± 0.09 mV (Figure S5a,d). 212.6 ± 2.04 nm ila 243.1 ± 0.60 nm

### 3.3. TEM Analysis Results

**Figure 1:**
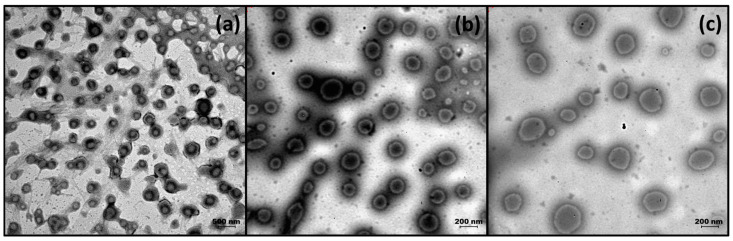
TEM image of EO-loaded PLGA nanoparticles; (**a**) OEO-loaded PLGA nanoparticles (magnitude 20 k); (**b**) CEO-loaded PLGA nanoparticles; and (magnitude 40 k) (**c**) OEO-CEO-loaded PLGA nanoparticles (magnitude 40 k).

Morphological analysis of OEO, CEO-loaded PLGA, and OEO-CEO-loaded PLGA nanoparticles was performed using TEM [56,57,58]. TEM images of OEO, CEO, and OEO-CEO-loaded PLGA nanoparticles are presented in Figure 1a–c, respectively. When the results obtained from the TEM analysis were examined, it was determined that the samples were homogeneously distributed, and the particle morphologies were spherical [59,60,61].

### 3.4. Stability Results of EO-Loaded PLGA Nanoparticles

The DLS method was used to determine whether there was any change in the physicochemical properties of the nanoparticles after lyophilization. The three-month stability test results of OEO, CEO and CEO-OEO-loaded PLGA nanoparticles under three different storage conditions (5 ± 3 °C, 25 ± 2 °C 60% RH, 40 ± 2 °C 75% RH) are presented in Table 3.

### 3.5. Determination of Encapsulation Efficiency and Loading Capacity

The encapsulation efficiency of OEO-loaded PLGA and CEO-loaded PLGA nanoparticles was calculated as 85.42% and 66.25%, respectively. The encapsulation efficiency of OEO-CEO-loaded PLGA nanoparticles was calculated as 85.14% for OEO and 66.28% for CEO. The loading capacity of OEO-loaded PLGA and CEO-loaded PLGA nanoparticles was calculated as 59.12% and 52.47%, respectively. The loading capacity of OEO-CEO-loaded PLGA nanoparticles was calculated as 33.75% for OEO and 15.54% for CEO.

### 3.6. In Vitro Release Profile

**Figure 2:**
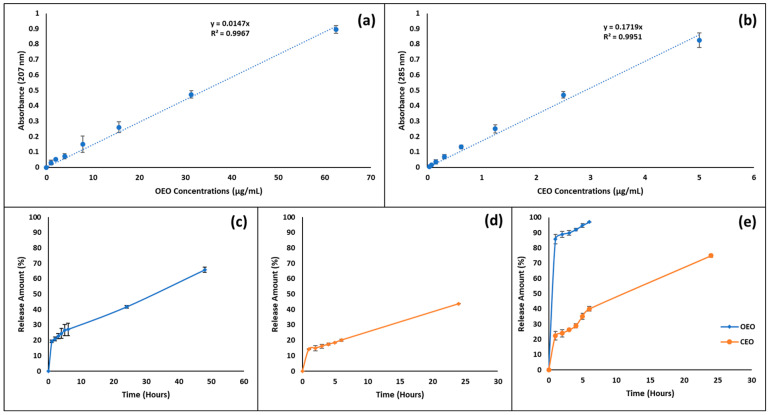
Calibration curves of Eos and in vitro release profile of EO-loaded PLGA nanoparticles; (**a**) the calibration curve of OEO; (**b**) the calibration curve of CEO; (**c**) OEO-loaded PLGA nanoparticles; (**d**) CEO-loaded PLGA nanoparticles; and (**e**) OEO-CEO-loaded PLGA nanoparticles (n = 3).

For the in vitro release study, the EO amounts in the samples taken from the release medium were calculated from the standard curve (Figure 2a,b) and the release profile of EO-loaded nanoparticles was evaluated as a function of time (Figure 2c–e). As seen in the results, OEO-loaded PLGA nanoparticles released 65.72% ± 0.57% OEO within 48 h (Figure 2c). CEO-loaded PLGA nanoparticles released 44.27% ± 0.72% CEO within 48 h (Figure 2d). OEO-CEO-loaded PLGA nanoparticles released 96.92% ± 1.11% OEO within 6 h and 74.94% ± 4.66% CEO at the end of 24 h (Figure 2e).

### 3.7. Ames Test Results

In our study, TA98 and TA100 mutant strains of *S. typhimurium* were used to evaluate the genotoxicity of OEO, CEO and OEO-CEO. Simultaneously with the evaluation of the geneotoxicity of essential oils, the genotoxicity of EO-loaded PLGA nanoparticles was also evaluated. Genotoxicity results of OEO-loaded PLGA nanoparticles and OEO are presented in Table 4, genotoxicity results of CEO-loaded PLGA nanoparticles and CEO are presented in Table 5 and genotoxicity results of OEO-CEO PLGA-loaded nanoparticles and OEO-CEO are presented in Table 6.

### 3.8. MIC Test Results

The effects of CEO, OEO, CEO-OEO, blank PLGA nanoparticles, CEO- or OEO-loaded PLGA nanoparticles and CEO-OEO-loaded PLGA nanoparticles on *S. mutans* and *L. casei* were investigated using the MIC method. While 1 mg/mL concentration was used for blank and loaded PLGA nanoparticles in the MIC method, EOs were applied according to the amount added to the synthesis. The results of the MIC study are given in Table 7.

### 3.9. In Silico Molecular Docking Results

The main active component of CEO that we used in our study is cinnamaldehyde (3-phenyl-2-propenal phenol; C_9_H_8_O), a phenylpropanoid. The amount of cinnamaldehyde in the essential oil obtained from the shells was determined to be 70.26%. The 3D molecular structure of the active ingredient cinnamaldehyde was obtained from PubChem (PubChem ID: 637511) and optimized at the PM3 theory level [62] using the Gaussian 09 Version 9.5 Package Program [63] and the spatial geometry where it can be found in the most stable, most effective and most active form was obtained. The second EO we used in our study is OEO. According to the oil analysis findings, although 15 different components were detected in the oil, the major one among them was D-limonene (97.30%). The 3D structure of D-limonene (C_10_H_16_) was obtained using PubChem (PubChem ID: 440917) and optimized at PM3 theory level [62] using the Gaussian 09 Version 9.5 Package Program [63] package program to obtain the most active and stable geometry. These two optimized active components were used as active ligands for molecular docking analysis. In addition, the inhibitory characteristics of carvacrol, one of the main components of oregano oil, which is widely known for its antibacterial effects, against *S. mutans* and *L. casei* were also investigated at the molecular level by molecular docking analysis and compared with cinnamaldehyde and D-limonene. Carvacrol was also optimized at the same theory level as other ligands withdrawn from PubChem (PubChem ID: 10364).

The antibacterial properties of these active ingredients, namely their inhibitory activities against *S. mutans* and *L. casei*, were analyzed and evaluated by in silico molecular docking method. Schrödinger program Maestro 11.4 Glide module was used in molecular docking studies. Our active receptors for molecular docking analysis are *S. mutans* and *L. casei*, and the three-dimensional structures of the bacteria were determined using the protein data bank.

**Figure 3:**
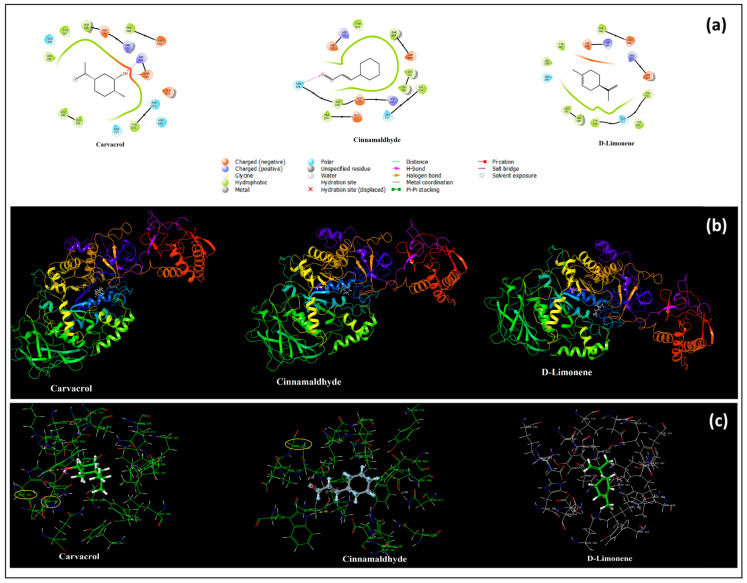
Active ligands bound to *S. mutans* (PDB:3AIC) and their 2D interactions (**a**); binding conformations of docked active ligands (**b**); hydrogen bond interactions of docked active ligands in the active binding pocket of the bacteria (**c**).

**Figure 4:**
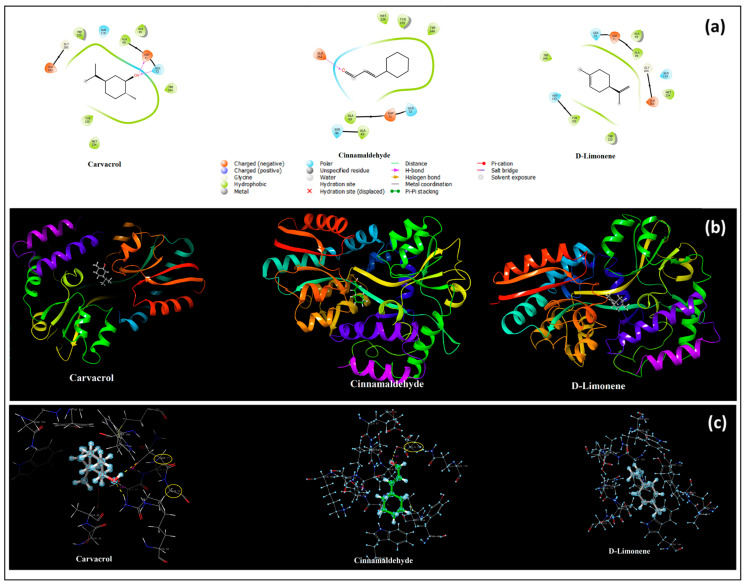
Active ligands bound to *L. casei* (PDB:5MTU) and their 2D interactions (**a**); binding conformations of docked active ligands (**b**); hydrogen bond interactions of docked active ligands in the active binding pocket of the bacteria (**c**).

Bacterial codes (PDB ID: 3AIC and 5MTU) were selected in the protein data bank, respectively. It was determined that there were sometimes missing residues in the receptor structure obtained from the protein data bank; such a deficiency was corrected using the SWISS-MODEL server. Using the “receptor preparation wizard” in the Schrödinger Glide module, all water and other molecular structures, such as ions, were removed from the structure, and polar hydrogen atoms were added to the receptor. Bond arrangements were determined, charges were determined with PROPKA at neutral pH, and the selected receptor was selected and optimized by approaching heavy atoms with an RMSD value of 0.3 Å. In addition, using the OPLS force field with the “LigPrep” tool in the Glide module of this program, cinnamaldehyde, the main active component of Cinnamon (*Cinnamomum cassia*) peel essential oil. D-limonene, the main active component of Orange (*Citrus sinensis*) peel EO, was used to form conformers with different geometries at neutral pH. All these conformers were used for docking. Grids were created to determine the active binding sites of the receptors, namely the most active binding sites where Cinnamaldehyde and D-limonene ligands would bind to *S. mutans* and *L. casei* and inhibit the bacteria, and the most active ligand–receptor binding sites were determined. After the grid process, molecular docking analysis was applied to determine the binding affinities, binding sites, binding mechanisms (with the help of which types of bonds the binding occurs) of cinnamaldehyde and D-limonene couplings to *S. mutans* and *L. casei* and their inhibitory efficiencies against *S. mutans* and *L. casei* were compared among themselves. Using the molecular docking analysis method, which is an in silico molecular modeling method, the antibacterial activities of cinnamon (*Cinnamomum cassia*) peel EO and orange (*Citrus sinensis*) peel EO were determined and revealed in three dimensions with atomic interactions and thus, the different inhibition mechanisms of different EOs on different bacteria, that is, their antibacterial activities, were determined by simulating them for the first time in a computer environment, and the comparative results were presented in Figure 3 and Figure 4 and Table 8.

Firstly, the crystal structure of our glucansucrase (GSase) receptor from *S. mutans* (PDB:3AIC), an important agent in the pathogenesis of dental caries, was made suitable for molecular docking analysis, and all ligands were docking to the active binding site of the bacteria. Figure 3a shows the interactions of carvacrol, cinnamaldehyde and D-limonene molecules in the binding site. Carvacrol was selected as the control group in Table 8. In addition, the interactions of cinnamaldehyde and D-limonene molecules, which are the subject of our study, in the binding region to *S. mutans* and *L. casei* are given in comparison with carvacrol.

As can be seen from Table 8 and Figure 3a, carvacrol, which is the most well-known and frequently used molecule, was selected as the control molecule in our study, and it was determined that carvacrol binds to *S. mutans* from the same binding site as cinnamaldehyde and D-limonene. When the docking score energies were compared, it was determined that the strongest binding was to carvacrol with −5.34 kcal/mol, and the second strongest binding was to cinnamaldehyde, the main active component of cinnamon bark EO, with −3.69 kcal/mol. D-limonene, the main active component of orange peel EO, was observed to bind with a binding energy of −3.235 kcal/mol. Carvacrol provided this stable binding by binding to the positively and negatively charged residues ASP345 (1.63) and HIS344 (1.89) with two hydrogen bonds with its -OH group. Cinnamaldehyde provided this binding with a hydrogen bond interaction between the oxygen atom in the tail and the polar ASN238(2.08) in the binding region. D-limonene, which has no donor or acceptor atom, managed to take place in this binding pocket without any hydrogen bond interaction. The conformations of the active ligands (carvacrol, cinnamaldehyde, and D-limonene) docked to *S. mutans* (PDB:3AIC) in the binding pocket of the bacteria are given in Figure 3b. In addition, the hydrogen bond interactions formed by these active ligands in the active binding pocket of the bacteria are shown in Figure 3c.

The inhibitory activity of the main active ingredients contained in CEO and OEO against *L. casei*, which causes dental caries, was also analyzed by molecular docking calculation. The crystal structure of *L. casei* at 1.00 Å resolution (PDB:5MTU) was selected as the 2nd receptor structure. The active binding site of *L. casei* and the binding of active compounds to this site are shown in Figure 4a. As can be seen from Figure 4a and Table 8, carvacrol provided a stable binding with the two hydrogen bonds made by its -OH group with the negatively charged aspartic acid (71) and polar glutamine (72) and the resulting docking energy was calculated as −5.81 kcal/mol.

Cinnamaldehyde settled into this pocket by making a hydrogen bond (1.91) with the negatively charged glutamic acid (262). Although D-limonene did not make any bonds, it made a more stable and stronger binding (−4.22 kcal/mol) than cinnamaldehyde. Cinnamaldehyde bound to *S. mutans* more stably than D-limonene, while D-limonene bound to *L. casei* more strongly than cinnamaldehyde. The conformations of the active ligands (carvacrol, cinnamaldehyde, and D-limonene) bound to *L. casei* in the binding pocket of the bacteria are shown in Figure 4b. In addition, the hydrogen bond interactions formed by these active ligands in the active binding pocket of the bacteria are shown in Figure 4c.

### 3.10. DNA Binding Results

**Figure 5:**
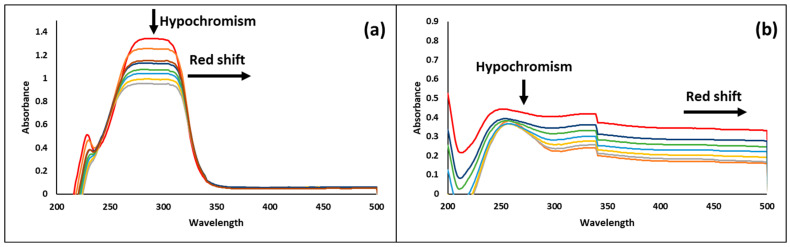
Absorption spectrum of CEO (**a**) and OEO (**b**) in the presence of increasing amounts of CT-DNA. The red line represents the spectrum in the absence of CT-DNA, while other lines correspond to spectra in the presence of increasing concentrations of CT-DNA.

UV–Vis absorption spectrophotometry is a valuable technique for investigating the interaction between drug molecules and DNA [54,64]. Therefore, within the scope of our study, the DNA binding activity of OEO and CEO was evaluated and shown in Figure 5. As a result of the interaction of the CEO with the successively added CT-DNA, 29.09% hypochromism and a 6 nm red shift were observed in UV–Vis absorption at 288 nm (Figure 5a). On the other hand, UV–Vis spectra of OEO showed that the interaction of OEO with CT-DNA resulted in a red shift of 6 nm at 251 nm and a hypochromic effect of 17.60% (Figure 5b).

### 3.11. DNA Cleavage Results

**Figure 6:**
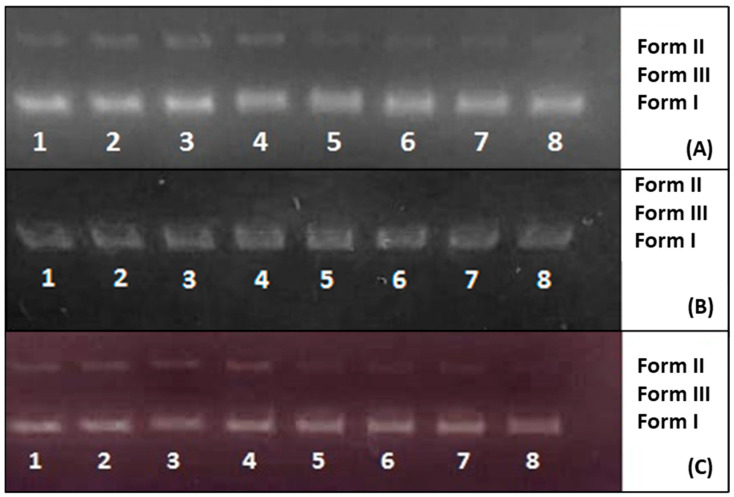
Hydrolytic DNA cleavage activity of EOs and loaded PLGA nanoparticles: (**A**) OEO and OEO-loaded PLGA nanoparticles (1: 0.125 mg/mL OEO-PLGA NP + DNA, 2: 0.250 mg/mL OEO-PLGA NP + DNA, 3: 0.50 mg/mL OEO-PLGA NP + DNA, 4: 1 mg/mL OEO-PLGA NP + DNA, 5: 0.10 mg/mL OEO + DNA, 6: 0.20 mg/mL OEO + DNA, 7: 0.40 mg/mL OEO + DNA, 8: 0.80 mg/mL OEO + DNA); (**B**) CEO and CEO-loaded PLGA nanoparticles (1: 0.125 mg/mL CEO-PLGA NP + DNA, 2: 0.250 mg/mL CEO-PLGA NP + DNA, 3: 0.50 mg/mL CEO-PLGA NP + DNA, 4: 1 mg/mL CEO-PLGA NP + DNA, 5: 0.10 mg/mL CEO + DNA, 6: 0.20 mg/mL CEO + DNA, 7: 0.40 mg/mL CEO + DNA, 8: 0.80 mg/mL CEO + DNA); (**C**) OEO-CEO and OEO-CEO PLGA NP (1: 0.125 mg/mL OEO-CEO-PLGA NP + DNA, 2: 0.250 mg/mL OEO-CEO-PLGA NP + DNA, 3: 0.50 mg/mL OEO-CEO-PLGA NP + DNA, 4: 1 mg/mL OEO-CEO-PLGA NP + DNA, 5: 0.10 mg/mL OEO-CEO + DNA, 6: 0.20 mg/mL OEO-CEO + DNA, 7: 0.40 mg/mL OEO-CEO + DNA, and 8: 0.80 mg/mL OEO-CEO + DNA).

Gel images of EOs and EO-loaded PLGA nanoparticles are shown in Figure 6. The results showed that OEO and OEO-loaded PLGA nanoparticles (Figure 6A) and OEO-CEO and OEO-CEO-loaded PLGA nanoparticles (Figure 6C) had only hydrolytic cutting activity. Hydrolytic cleavage reduced some supercoiled DNA to an open circular form (Form II). On the other hand, no cleavage activity was observed in CEO and CEO-loaded PLGA nanoparticles (Figure 6B).

## 4. Discussion

To evaluate the average particle size, PdI, and zeta potential of blank PLGA and EO-loaded PLGA nanoparticles in our work, the commonly used DLS method was used [43,61]. The results were compared with other studies in the literature. Priyadarshini et al. (2018) synthesized clove oil-loaded PLGA nanoparticles for dental applications. They reported that blank nanoparticles had an average particle size of ∼127.84 ± 18.46 nm, while 10 and 25 mg oil-loaded nanoparticles had an average particle size of 165.37 ± 22.53 and 237.63 ± 13.68 nm, respectively [65]. Phuangkaew et al. (2022) reported that nanoparticles obtained from amphiphilic quaternized chitosan for the prevention of dental caries have a hydrodynamic diameter of ~100–300 nm [66]. Minhaco et al. (2023) synthesized curcumin-loaded PLGA nanoparticles for the treatment of endodontic biofilms and showed that blank and curcumin-loaded nanoparticles had an average particle size of 189.2 ± 13.630 and 247.6 ± 3.651 nm, respectively [67]. In this context, it was concluded that the nanoparticles synthesized in our study have a suitable size range for dental applications. On the other hand, PdI values below 0.2 represent a narrow particle size range and monodispersity [68]. Since the PdI value of the PLGA nanoparticles synthesized in our study was in the range of 0.032–0.078, it was concluded that they were monodisperse. Moreover, the zeta potential, which is used to measure the electrokinetic potential in colloidal systems [69], is an important parameter for determining both the behavior of drug delivery systems [70] and their colloidal stability [71]. Gursu et al. (2022) synthesized cinnamaldehyde-loaded PLGA nanoparticles. The results reported that the zeta potential values of these particles ranged from − 3.54 to − 3.86 mV under three different storage conditions, and they showed good stability as higher negative zeta potential values increased the stability of the nanoparticles [72]. Minhaco et al. (2023) synthesized curcumin-loaded PLGA nanoparticles to treat endodontic biofilms. They showed that blank and curcumin-loaded nanoparticles had zeta potential values of −0.935 ± 0.166 mV and −1.65 ± 0.355 mV, respectively [67]. In this context, it was concluded that the nanoparticles synthesized in our study have a high zeta potential value compared to these studies in the literature.

In the commercialization phase, stability is strategically important regarding product shelf life. In this context, average particle size, PdI, and zeta potential values were compared to evaluate the stability of OEO-loaded PLGA, CEO-loaded PLGA, and OEO-CEO-loaded PLGA nanoparticles after lyophilization. The results showed that the DLS analysis results for the first day, first month, second month, and third month were close to each other, and there was no change that would cause significant stability problems. PVA used in PLGA nanoparticle preparation is known as a steric stabilizer [73]. Here, steric stabilization is achieved by creating a repulsive force between the particles and droplets in the dispersion [74] and preventing van der Waals attraction between nanoparticles [75]. PVA used in the particle synthesis stage can be permanently bound to the nanoparticle surface thanks to the hydrophobic bond between partially hydrolyzed poly(vinyl acetate) groups and PLGA acetyl groups [76]. Therefore, it was concluded that the nanoparticle formulations produced within the scope of our study remained stable for a long time after the lyophilization process and could be a product with a long shelf life since steric stabilization was provided by PVA bound to its surface in addition to the zeta potential.

High-level entrapment of the active ingredient in polymeric nanoparticles is important for developing effective nanoformulations. In this context, encapsulation efficiency and loading capacity parameters are a strategic approach to determine how much of the active ingredient is trapped in the polymeric envelope [77]. Within the scope of our study, the encapsulation efficiency of the synthesized EO-loaded PLGA nanoparticles was carried out by the solvent extraction method [44]. The results obtained in our study are consistent with the values obtained in the literature. Iannitelli et al. (2011) determined the encapsulation efficiency and loading capacity of carvacrol-loaded PLGA nanoparticles as 26% and 21%, respectively [78]. Esfandyari-Manesh et al. (2013) synthesized PLGA nanoparticles loaded with anethole and carvone EOs by emulsification solvent evaporation and nanoprecipitation methods and determined that the highest encapsulation efficiency was 87.31 ± 5.84 and 68.21 ± 0.90, respectively, and the highest loading efficiency was 14.73 ± 0.82 and 13.64 ± 0.19, respectively [79]. The encapsulation efficiency of the developed Bergamot EO-loaded nanoparticles was found to be between 28% and 84% [39]. It was reported that PLGA nanoparticles loaded with *Cymbopogon citratus* EO had an encapsulation efficiency of 73.29 ± 8.96 [80]. PLGA-chitosan-folic acid nanoparticles containing *Artemisia vulgaris* L. EO were produced, and the encapsulation efficiency was reported to be 99.79% [43]. In another study, EOs obtained from *Boswellia sacra* oleo gum resin were encapsulated with PLGA-PCL nanoparticles and the encapsulation efficiency was reported to be in the range of 27.04% ± 1.08 to 80.59 ± 3.37% [81]. In this context, it was concluded that the EOs-loaded PLGA nanoparticles synthesized within the scope of our study have a high encapsulation efficiency and loading capacity. On the other hand, OEO-CEO-loaded PLGA nanoparticles’ encapsulation efficiency and loading capacity values of OEO are higher than those of CEO. Various parameters, such as polymer concentration in the oil phase, intrinsic viscosity, carboxylic terminal group, and molecular weight of polymers, greatly influence the encapsulation efficiency [82]. Here, more hydrophilic drugs have lower affinity to the polymer, and this is an important parameter that reduces encapsulation efficiency [83]. Based on our findings, the major components for OEO and CEO were determined to be D-limonene and cinnamaldehyde, respectively. Here, the limonene compound consisting of two isoprene units is completely hydrocarbon in structure and does not contain any polar groups [84]. On the other hand, cinnamaldehyde is a compound containing two unsaturated functional groups of aldehyde and carbon-carbon double bond [85], and this aldehyde group increases the polarity of the structure [86]. Moreover, D-limonene has higher solubility in water compared to cinnamaldehyde (13.8 mg/L [87] and 1.42 mg/mL [88], respectively, at 25 °C). In this context, it was understood that this difference was due to the hydrophilicity and polarity of the major components in EOs and the better diffusion of OEO into the PLGA polymer compared to CEO.

The release of EOs from the nanoparticle depends on several factors, such as capsule wall thickness, affinity for the active compound PLGA, diffusion of the active compound through the polymer matrix, polymeric erosion, PLGA swelling and degradation [89]. These parameters reveal the reason why the release profiles of EO-loaded PLGA nanoparticles in the literature are different from each other. Zhu et al. (2019) synthesized thymol-loaded PLGA microparticles and reported releasing 50% of thymol in 72 h [90]. These results were closer to the amount of OEO (65.72 ± 0.57% within 48 h) released from PLGA nanoparticles loaded with OEO and CEO (44.27 ± 0.72% within 48 h) released from PLGA nanoparticles loaded with CEO. In another study with PLGA nanoparticles loaded with carvacrol, it was reported that 95% of carvacrol was released within 24 h [78]. On the other hand, OEO-CEO-loaded PLGA nanoparticles released almost all of the OEO in 6 h, while the CEO released 74.94 ± 4.66% in 24 h. Manesh et al. (2013) developed anethole and carvone EO-loaded PLGA nanoparticles and reported that approximately 41% of anethole in 9 days and 50% of carvone in 4 days were released from PLGA nanoparticles [79]. In this study, they reported that the release profile they obtained showed slow-release characteristics and that the diffusion of the loaded EOs occurred when the loaded essential oils passed through the PLGA polymer chains in the nanoparticle-controlled release system they prepared and passed from the polymeric matrix to the external environment. When the results we obtained in our study were evaluated, it was observed that a slow release profile similar to the literature was obtained when the release profile for OEO-loaded PLGA and CEO-loaded PLGA nanoparticles was examined. However, OEO-CEO-loaded PLGA nanoparticles determined that the release profile obtained from encapsulating two EOs with the polymer has a faster release profile than the PLGA nanoparticle-controlled system with a single EO load (Figure 2e). The main component of CEO, cinnamaldehyde [86], has a relatively polar property due to the aldehyde group it contains in its structure [85]. Compounds containing aldehyde groups can form classical (OH····O) and weak (CH····O) hydrogen bonds with compounds containing carboxyl groups [91]. Moreover, the carbonyl units in the PLGA structure can form hydrogen bonds [92]. Therefore, CEO encapsulated in OEO-CEO-loaded PLGA nanoparticles may interact with the polymer and be released slower compared to OEO. On the other hand, since its main component, limonene does not contain a polar group [84], OEO has a relatively more nonpolar property than CEO. Therefore, encapsulated CEO may partially polarize the polymer matrix and cause OEO-matrix compatibility to decrease, resulting in faster release of OEO, which has a higher nonpolar property.

Medicinal plants have been widely used in the development of new drugs from the past to the present. However, the use of plants in drug development causes safety concerns during preclinical evaluation. Genotoxicity analyses are extremely important in preclinical evaluation [93]. Therefore, the Ames test, one of the main tools of genetic toxicology, has become part of a series of preclinical tests for the detection of the genotoxic potential of medicinal plants [94,95,96]. When the concentrations of EOs used were evaluated by statistical analysis and Mortelman and Zeiger, it was determined that they had genotoxic effects on TA98 and TA100 mutant strains (*p* < 0.05). In addition, at some concentrations of EOs, the number of colonies returned on the plate was very low. This indicates that the EO concentrations used are highly toxic [48]. It was determined that EO-loaded PLGA nanoparticles did not have a genotoxic effect on *S. typhimurium* TA98 and TA100 mutant strains according to both Mortelman and Zeiger and statistical analysis results (*p* > 0.05). Our results clearly show that encapsulation of EOs with PLGA abolishes the genotoxicity of EOs.

Antibacterial activity tests are important methods for analyzing substances that can be used against disease-causing microorganisms [97]. In this study, the effects of CEO, OEO, CEO-OEO, CEO- or OEO-loaded PLGA nanoparticles, CEO-OEO-loaded PLGA nanoparticles, and blank PLGA nanoparticles on *L. casei* and *S. mutans*, which negatively affect dental health, were determined. Based on the MIC results, both bacteria were found to be highly sensitive to CEO. Studies in the literature have determined that CEO is effective on both gram-positive and gram-negative bacteria [98,99,100]. The active compound, cinnamaldehyde, is responsible for antibacterial activity, and the acrolein group (α,β-unsaturated carbonyl part) in this molecule has an important place for activity [101]. Despite this, CEO-loaded PLGA nanoparticles were found to have a lower effect on bacteria than CEO. This was interpreted as due to the slow and controlled release of CEO from CEO-loaded PLGA nanoparticles [89]. When the effectiveness of OEO on *L. casei* and *S.mutans* was examined, no activity was observed against *S. mutans*. Pattnaik et al. (2010) evaluated the effectiveness of cinnamon oil, peppermint oil, cardamom oil and orange oil as antibacterial agents. The study found that orange oil was ineffective for gram-positive bacteria [102]. Despite this, OEO was found effective on *L. casei* and inhibited bacterial growth in MIC method. Moreover, it was found that OEO-loaded PLGA nanoparticles did not exhibit bactericidal effects on *L. casei* and *S. mutans* bacteria. On the other hand, *L. casei* and *S. mutans* were susceptible to CEO-OEO, with the antibacterial effect of CEO contributing significantly. Accordingly, it was determined that CEO-OEO-loaded PLGA nanoparticles were effective on these bacteria. When the minimum inhibitory concentration determined in the MIC method was examined, it was seen that CEO-OEO-loaded PLGA nanoparticles were effective on *L. casei* and *S. mutans* and had the same bactericidal effect. These findings were evaluated based on other studies in the literature. Limonene was determined to be the major component in EOs obtained from *Citrus reticulata* Blanco peels, and a synergistic effect was observed on methicillin-resistant/susceptible *S. aureus* as a result of the combined application of gentamicin/limonene and gentamicin/EOs [103]. Application of gentamicin/D-limonene in combination showed a synergistic effect on multidrug-resistant *S. aureus* and *E. coli* strains, and MIC values decreased from 13.71 μg/mL to 4 μg/mL and from 30 μg/mL to 20.1 μg/mL, respectively [104]. Echeverry-Chica et al. (2020) determined that nanoemulsions containing D-limonene-functionalized Ag nanoparticles showed a higher antibacterial effect compared to emulsions containing Ag nanoparticles or limonene alone [105]. Motelica et al. (2023) reported that ZnO nanoparticles loaded with different EOs showed a higher inhibition zone compared to ZnO nanoparticles or EOs applied alone. They attributed this to sensitization by EOs, making the bacterial cell less resistant to ZnO nanoparticles [106]. Motelica et al. (2024) developed hydroxyethylcellulose-based composite materials with ZnO nanoparticles, CEO-loaded mesoporous silica nanoparticles, and their combination. The combined material showed higher antibacterial activity against *S. aureus* and *E. coli* strains than CEO-loaded mesoporous silica nanoparticles loaded composite alone, providing a synergistic effect [107]. Moreover, it is stated that the properties of the D-limonene compound, such as increasing the permeability of the antimicrobial substance or its lipophilic properties passing through the cell wall and changing the permeability of the cell membrane, may be related to this synergistic effect [108]. In this context, our study has shown that the combined use of two EOs has a synergistic effect with OEO or CEO, thanks to limonene, the major component in its content, and is effective on both gram-positive and gram-negative bacteria.

Moreover, in our in silico results, it was determined that cinnamaldehyde, the main active component of CEO, and D-limonene, the main active component of OEO, bind to *S. mutans* and *L. casei* and that this binding occurs via the binding site to which carvacrol is found in the content of thyme oil, also binds, and that hydrogen bonds provide this binding. Moreover, it was determined by molecular docking analysis that the binding of cinnamaldehyde to *S. mutans*, i.e., its inhibition, was greater than D-limonene considering the docking score energy. However, the opposite was true for the binding and inhibition of *L. casei*, with D-limonene providing a more stable binding. It was observed as a result of this study that the main active ingredients of both EOs could be at least as effective as carvacrol in the content of oregano oil against these bacteria.

In our study, DNA binding activity of OEO and CEO was evaluated, and it was determined that both CEO and OEO showed a 6 nm red shift and 29.09% and 17.60% hypochromic effect, respectively, as a result of their interaction with CT-DNA. In spectral effects, the blank π* orbital of the molecule pairs with the π* orbital of the DNA base pairs, resulting in an energy decrease and a lowering of the π-π* transition energy. This is detected by the red shift of the absorption in the molecular DNA interaction. At the same time, the blank π* orbital is partially filled with electrons to reduce the probability of transition, resulting in hypochromism [109,110]. Hypochromism and red-shift are considered to be indicators of intercalative binding of compounds with DNA [38,111]. Compounds with clinical potential may interact with DNA by intercalation or groove linking [112]. In this context, the results obtained show that OEO and CEO exhibit intercalative interaction with DNA and, therefore, may be effective agents for antibacterial activity.

DNA cleavage activity of OEO, CEO, CEO-OEO, OEO-loaded PLGA, CEO-loaded PLGA, and CEO-OEO-loaded PLGA nanoparticles was determined using pBR322 plasmid DNA by agarose gel electrophoresis [113,114,115]. The main targets in DNA cutting are the phosphodiester bond, deoxyribose sugar, or nucleobases of DNA. It is possible to cut these important parts of DNA by hydrolytic or oxidative means. While hydrolytic cleavage occurs at the phosphodiester bonds of DNA, oxidative cleavage occurs at the deoxyribose sugar or nucleobases of DNA [116]. In the study, pBR322 plasmid DNA was used for DNA-cutting activity. Circular plasmid DNA migrates relatively quickly in gel electrophoresis (Form I). If cutting occurs in a single strand of plasmid DNA, the supercoiled state of the plasmid relaxes and transforms into Form II. If both strands are cleaved, Form III is formed, a linear form that migrates between Form I and Form II [117]. When these results were examined, it was interpreted that the reason for the lack of cleavage activity in CEO-loaded PLGA nanoparticles could be due to the DNA cleavage activity test system being completed in a short time of 3 h and CEO being released slowly from the particle (44.27 ± 0.72% in 48 h) and not showing any activity. On the other hand, it was thought that the cleavage activity of OEO-loaded PLGA nanoparticles and OEO-CEO-loaded PLGA nanoparticles could be due to the rapid release.

## 5. Conclusions

EOs are products formed from secondary metabolites of plants and have therapeutic properties such as antibacterial, antifungal, and antioxidant. Moreover, research on using EOs as preventive or therapeutic agents for oral diseases, including dental caries, has gained momentum. In this context, in our study, OEO, CEO, and CEO-OEO-loaded PLGA nanoparticles were synthesized in order to develop a controlled drug delivery system for the prevention and treatment of dental caries, and the synergistic effect of two different EOs was comparatively investigated. In our study, it was found that the main phytochemical of OEO was limonene, with a rate of 97.309%, and the main components of CEO were cinnamaldehyde, with a rate of 70.267%. EOs-loaded PLGA nanoparticles had appropriate average particle size, PdI value, and zeta potential values. They were observed to have homogeneously distributed and spherical morphology as a result of DLS and TEM analysis. Moreover, it was determined after a 3-month study that the nanoparticle formulations remained stable for a long time after the lyophilization process. The synthesized nanoparticles had high encapsulation efficiency and loading capacity values and were able to release the active ingredient in a long-term and controlled manner. The Ames test determined that the EOs used in our study had a genotoxic effect, but this effect was eliminated as a result of their encapsulation into PLGA nanoparticles. Antibacterial experiments showed that CEO-loaded PLGA nanoparticles were effective on *S. mutans* bacteria. In contrast, OEO-CEO-loaded PLGA nanoparticles were effective on *L. casei* and *S. mutans*, carrying the antibacterial properties of both EOs. DNA binding assays showed that OEO and CEO exhibit intercalative interaction with DNA and therefore could be effective agents for antibacterial activity. DNA cutting assay results showed that OEO-loaded PLGA nanoparticles and OEO-CEO-loaded PLGA nanoparticles had hydrolytic cutting activity. Moreover, it was determined through molecular docking studies that the main active ingredients of each EO could be at least as effective as carvacrol in oregano oil on *L. casei* and *S. mutans* bacteria. In conclusion, it was evaluated that the CEO-OEO-loaded PLGA nanoparticles synthesized in our study were effective on both gram-negative and gram-positive bacteria with synergistic effects, had effective antibacterial properties for use in dental caries prophylaxis, and were a suitable candidate for the development of nanoformulations that could be used as mouthwash in the future.

This study successfully demonstrated the effect of encapsulation of EOs with PLGA nanoparticles and controlled release mechanisms. Release kinetics reveal the potential to increase the bioavailability of EOs. In addition, the use of different polymer shells (except for poly(lactic-co-glycolic acid) (PLGA), e.g., polycaprolactone (PCL), poly(lactic acid) (PLA), cellulose, and chitosan) in nanoparticle designs will be evaluated. The use of these alternative polymers has the potential to improve the release profiles and bioavailability of EOs. Our study’s release values of EOs obtained from PLGA nanoparticles vary according to the time and active substance. It is aimed for the formulations planned to be obtained using other polymers to provide maximum effectiveness by providing 100% release in 12 h. In line with the findings of our study, the lyophilized nanoparticles in single-use sachets are planned to be prepared immediately before use as a mouthwash product. Our study findings also indicate the potential of EOs to be used in toothpaste formulations, in addition to their use as mouthwashes. Such formulations may provide long-term protection against tooth decay, plaque formation, and bad breath due to the controlled release of EOs. Our future studies aim to investigate and optimize the effectiveness of these formulations in different oral health applications.

## Figures and Tables

**Table 1:** Chemical components of OEO.

Components	Amount (%)
Alpha Pinene	0.473
Beta Pinene	0.346
Myrcene	1.694
Limonene	97.309
Aromadendren	0.179

**Table 2:** Chemical components of CEO.

Components	Amount (%)
Alpha Pinene	0.790
Camphene	0.217
Benzaldehyde	0.263
Beta Pinene	0.213
Phellandrene	0.318
Cymene	1.916
Limonene	0.925
1.8-Cineole	1.941
Linalool	2.120
Terpinene-4-ol	0.538
Alpha Terpineol	1.331
Cinnamaldehyde	70.267
Eugenol	5.220
Caryophyllene	0.316
Cinnamyl Acetate	10.581
Eugenyl Acetate	0.821
Caryophyllene Oxide	0.132
Beznyl Benzaoate	1.705

**Table 3:** Stability results of EOs loaded with PLGA nanoparticles after lyophilization (n = 3).

NPs	Storage Conditions	DLS Analysis Parameters	Day 1	1st Month	2nd Month	3rd Month
OEO-loaded PLGA nanoparticles	5 ± 3 °C	Size (nm)	232.6 ± 2.95	203.6 ± 4.04	190.2 ± 3.46	189.3 ± 3.27
		PdI	0.196 ± 0,06	0.142 ± 0.04	0.152 ± 0.02	0.134 ± 0.01
		Zeta (mV)	−7.44 ± 0.36	−9.23 ± 0.07	−8.59 ± 0.57	−9.84 ± 0.39
	25 ± 2 °C 60% RH	Size (nm)	232.6 ± 2.95	202 ± 3.29	186.1 ±0.32	185.6 ± 1.97
		PdI	0.196 ± 0,06	0.143 ± 0.02	0.122 ± 0.01	0.125 ± 0.05
		Zeta (mV)	−7.44 ± 0.36	−7.81 ± 0.56	−9.84 ± 0.24	−10.4 ± 0.17
	40 ± 2 °C 75% RH	Size (nm)	232.6 ± 2.95	215.4 ± 4.69	194.3 ± 2.26	192.1 ± 2.58
		PdI	0.196 ± 0,06	0.126 ± 0.04	0.090 ± 0.01	0.183 ± 0.01
		Zeta (mV)	−7.44 ± 0.36	−9.85 ± 0.16	−11.66 ± 0.25	−8.42 ± 0.73
CEO-loaded PLGA nanoparticles	5 ± 3 °C	Size (nm)	191.1 ± 1.06	198.9 ± 2.64	188.5 ± 2.02	192.4 ± 2.82
		PdI	0.132 ± 0.02	0.183 ± 0.02	0.112 ± 0.03	0.106 ± 0.02
		Zeta (mV)	−5.20 ± 0.04	−12.1 ± 0.80	−9.97 ± 0.39	−10.56 ± 0.46
	25 ± 2 °C 60% RH	Size (nm)	191.1 ± 1.06	189.2 ± 1.28	191.7 ± 3.67	190.2 ± 1.91
		PdI	0.132 ± 0.02	0.144 ± 0.03	0.096 ± 0.01	0.146 ± 0.02
		Zeta (mV)	−5.20 ± 0.04	−10.66 ± 0.30	−10.8 ± 0.55	−11 ± 0.26
	40 ± 2 °C 75% RH	Size (nm)	191.1 ± 1.06	228.3 ± 1.92	188.7 ± 4.41	190.4 ± 1.70
		PdI	0.132 ± 0.02	0.214 ± 0.02	0.110 ± 0.02	0.206 ± 0.02
		Zeta (mV)	−5.20 ± 0.04	−7.61 ± 0.34	−12.8 ± 0.36	−9.97 ± 0.38
OEO-CEO-loaded PLGA nanoparticles	5 ± 3 °C	Size (nm)	211.8 ± 1.65	187.63 ± 2.73	243.1 ± 2.65	213 ± 1.76
		PdI	0.152 ± 0.01	0.171 ± 0.01	0.172 ± 0.09	0.122 ± 0.03
		Zeta (mV)	−4.79 ± 0.03	−9 ± 0.85	−6.37 ± 0.45	−8.25 ± 0.36
	25 ± 2 °C 60% RH	Size (nm)	211.8 ± 1.65	170.9 ± 0.65	230.3 ± 1.64	177.6 ± 0.32
		PdI	0.152 ± 0.01	0.118 ± 0.02	0.122 ± 0.04	0.105 ± 0.02
		Zeta (mV)	−4.79 ± 0.03	−8.42 ± 0.99	−7.23 ± 0.28	−7.50 ± 0.39
	40 ± 2 °C 75% RH	Size (nm)	211.8 ± 1.65	176.6 ± 2.30	225.9 ± 2.09	185.2 ± 1.92
		PdI	0.152 ± 0.01	0.075 ± 0.03	0.138 ± 0.05	0.144 ± 0.06
		Zeta (mV)	−4.79 ± 0.03	−7.88 ± 0.28	−8.42 ± 0.73	−6.82 ± 0.26

**Table 4:** Genotoxicity results of OEO and OEO-loaded PLGA nanoparticles.

Treatment	Concentrations (mg/mL)	Revertant Colonies/Plate	
		TA98 Mean ± SD	TA98 Mean ± SD
OEO	0.10	5.6 ± 2.08 *	45 ± 5.0 *
	0.20	8.3 ± 1.52 *	37 ± 2.0 *
	0.40	274 ± 7.93 *	39.66 ± 2.51 *
	0.80	606 ± 4.58 *	25.33 ± 4.93 *
OEO-Loaded PLGA Nanoparticles	0.125	26 ± 2.64	210 ± 4.58
	0.25	28.6 ± 1.52	226 ± 23.2
	0.50	22.6 ± 1.54	229.6 ± 6.65
	1.00	24.3 ± 3.05	235 ± 15.0
Positive Control (NPD)	0.005	847.3 ± 2.52 *	
Positive Control (SA)	0.0005		1242 ± 21.2 *
Negative Control (Water)		25 ± 3.60	191 ± 8.51
Negative Control (Ethanol)		27.6 ± 2.51	188 ± 3.60
Spontaneous Control		24 ± 1.00	188.66 ± 3.51

An asterisk (*) indicates that the difference in the mean number of returned colonies between the negative control and treatment groups was significant at the *p* < 0.05 level. SA: sodium azide; NPD: 4-Nitro-o-phenylenediamine.

**Table 5:** Genotoxicity results of CEO and CEO-loaded PLGA nanoparticles.

Treatment	Concentrations (mg/mL)	Revertant Colonies/Plate	
		TA98 Mean ± SD	TA98 Mean ± SD
CEO	0.10	6 ± 1.00 *	322 ± 7.54 *
	0.20	9.6 ± 2.08 *	83 ± 7.0 *
	0.40	7 ± 1.00 *	65 ± 13.74 *
	0.80	5 ± 2.00 *	6 ± 1.0 *
CEO-Loaded PLGA Nanoparticles	0.125	31.6 ± 3.51	200.6 ± 2.08
	0.25	26 ± 6.00	195.6 ± 5.13
	0.50	32 ± 2.64	209.3 ± 9.01
	1.00	31.3 ± 3.51	194.6 ± 25.3
Positive Control (NPD)	0.005	847.3 ± 2.52 *	
Positive Control (SA)	0.0005		1242 ± 21.2 *
Negative Control (Water)		25 ± 3.60	191 ± 8.51
Negative Control (Ethanol)		27.6 ± 2.51	188 ± 3.60
Spontaneous Control		24 ± 1.00	188.6 ± 3.51

An asterisk (*) indicates that the difference in the mean number of returned colonies between the negative control and treatment groups was significant at the *p* < 0.05 level. SA: Sodium azide; NPD: 4-Nitro-o-phenylenediamine.

**Table 6:** Genotoxicity results of OEO-CEO and OEO-CEO-loaded PLGA nanoparticles.

Treatment	Concentrations (mg/mL)	Revertant Colonies/Plate	
		TA98 Mean ± SD	TA98 Mean ± SD
OEO-CEO	0.10	7.00 ± 2.00 *	293 ± 3.21 *
	0.20	2.00 ± 1.00 *	508 ± 3.78 *
	0.40	5.33 ± 2.51 *	82 ± 2.51 *
	0.80	4.33 ± 2.08 *	9.66 ± 2.51 *
OEO-CEO-loaded PLGA nanoparticles	0.125	21.33 ± 3.51	246.6 ± 9.07
	0.25	24.33 ± 3.05	225 ± 5.0
	0.50	24.66 ± 2.30	228 ± 7.02
	1.00	23.66 ± 2.51	207.3 ± 2.08
Positive Control (NPD)	0.005	847.33 ± 2.52 *	
Positive Control (SA)	0.0005		1242 ± 21.2 *
Negative Control (Water)		25 ± 3.60	191 ± 8.51
Negative Control (Ethanol)		27.66 ± 2.51	188 ± 3.60
Spontaneous Control		24 ± 1.00	188.66 ± 3.51

An asterisk (*) indicates that the difference in the mean number of returned colonies between the negative control and treatment groups was significant at the *p* < 0.05 level. SA: sodium azide; NPD: 4-Nitro-o-phenylenediamine.

**Table 7:** The MIC results of CEO, OEO, CEO-OEO, CEO-OEO-loaded PLGA nanoparticles, CEO-OEO-loaded PLGA nanoparticles, and blank PLGA nanoparticles.

Samples	MIC (mg/mL)	
	*L. casei*	*S. mutans*
CEO-loaded PLGA NPs	-	0.25
OEO-loaded PLGA NPs	-	-
CEO-OEO-loaded PLGA NPs	0.5	0.5
CEO	0.40	0.20
OEO	0.80	-
CEO-OEO	0.20	0.10
Blank PLGA NPs	-	-

-; no activity.

**Table 8:** Analysis of the inhibitory activity of the main active ingredients in CEO and OEO on *S. mutans* and *L. casei*, which cause dental caries, by molecular docking calculation.

Selection of Key Ingredients in EOs	The Main Active Ingredient of CEO Is Cinnamaldehyde, a Phenylpropanoid			The Main Active Ingredient of OEO Is D-Limonene		
Receptors	*Streptococcus mutans PDB:3AIC*			*Lactobacillus casei PDB:5MTU*		
Ligands	Carvacrol (Supplement)	Cinnamaldehyde	D-Limonene	Carvacrol (Supplement)	Cinnamaldehyde	D-Limonene
*Docking score (Kcal/mol)*	−5.349	−3.693	−3.235	−5.818	−4.098	−4.221
*H bond interactions (Angstrom)*	ASP345(1.63) HIS344(1.89)	ASN238(2.08)	-	ASP71(1.96) GLN72(1.87)	GLU262(1.91)	-
*Salt bridge interaction*	-	-	-	-	-	-
*Pi-cation interaction*	-	-	-	-	-	-
*Hydrophobic* *residues*	LEU139, TYR367 ALA235, PHE664, TYR673, LEU191, LEU190	TYR673, PHE664, LEU191, LEU190, VAL714, ALA235, TRP274	TYR367, LEU139, LEU191, LEU190, TYR719, TYR673, VAL714, PHE664	ALA49, ALA69, TRP225, TRP344, TYR155, MET334	MET334, TYR155, TRP344, ALA69, ALA49	ALA49, ALA69, MET334, TRP225, TYR155, TRP344
*Polar residues*	GLN349, ASN671, ASN619, GLN717	ASN238, GLN717	GLN717, GLN349	GLN119, GLN72	GLN72, SER48	GLN119, GLN72, ASN153
*Positively charged* *residues*	ARG232, HIP344	ARG232, HIP344	ARG232, HIP344	-	-	-
*Negatively charged residues*	ASP234, ASP666, ASP345, GLU272	ASP234, ASP666, ASP345, GLU272	ASP234, ASP666, ASP345,	ASP71, GLH262	ASP71, GLH262	ASP71, GLH262
*Glycine*	-	-	-	GLY260	-	GLY260

## Data Availability

The raw data supporting the conclusions of this article will be made available by the authors on request.

## Supplementary Materials

The following supporting information can be downloaded at https://www.mdpi.com/article/10.3390/pharmaceutics17020167/s1: Table S1: GC-MS/FID conditions; Figure S1: GC-MS chromatogram of EOs; (a) OEO and (b) CEO; Figure S2. DLS analysis results: (a) average particle size, PdI, and (b) zeta potential graphics of blank PLGA nanoparticles; Figure S3. DLS analysis results: (a) average particle size, PdI, and (b) zeta potential graphics of OEO-loaded PLGA nanoparticles; Figure S4. DLS analysis results: (a) average particle size, PdI, and (b) zeta potential graphics of CEO-loaded PLGA nanoparticles; Figure S5. DLS analysis results: (a) average particle size, PdI, and (b) zeta potential graphics of OEO-CEO-loaded PLGA nanoparticles.
