# Supplementary material for: Production of Prophylactic Nanoformulation for Dental Caries and Investigation of Its Effectiveness by In Vitro and In Silico Methods
Source: Pharmaceutics. 2025 Jan 27;17(2):167. doi: 10.3390/pharmaceutics17020167 (PMC11859156; doi:10.3390/pharmaceutics17020167)
Supplement: Supplementary file 1 [file pharmaceutics-17-00167-s001.zip › pharmaceutics-3404799-supplementary.pdf]

Supplementary materials

1. GC-MS/FID Conditions

Table S1. GC-MS/FID conditions.

GC-MS/FID CONDITIONS	
System:	Agilent 7890B GC
Carrier Gas:	Helium
Flow Rate of Carrier Gas:	1,5 mL/min
Injector Temperature:	220 ^{0}C
Injection Volume:	1 μL
Split Ratio:	50:1
Column:	Agilent DB-Wax (60m x 0,25mm x 0,25 μm)
Oven Temperature Programme:	70^{0}C (15 min), 2^{0}C/min → 180^{0}C (5 min), 5^{0}C/min → 230^{0}C (15 min)
Detector:	FID (Flame Ionisation Detector)
Detector Temperature:	220 ^{0}C
FID Hydrogen:	30 mL/min
FID Air:	400 mL/min
Detector:	MS (Mass Spectrometer)
System:	5977E
Ion Source Temperature:	230 ^{0}C
Ionisation Mode:	Electron Impact Ionization (EI)
Electron Energy:	70 eV
Interface Temperature:	250^{0}C
Quadrupole Temperature:	150^{0}C
Mass Scan Range:	35-450 m/z
Identification:	Wiley 9-NIST 11 Mass Spectral Database

2. GC-MS Chromatogram of EOs

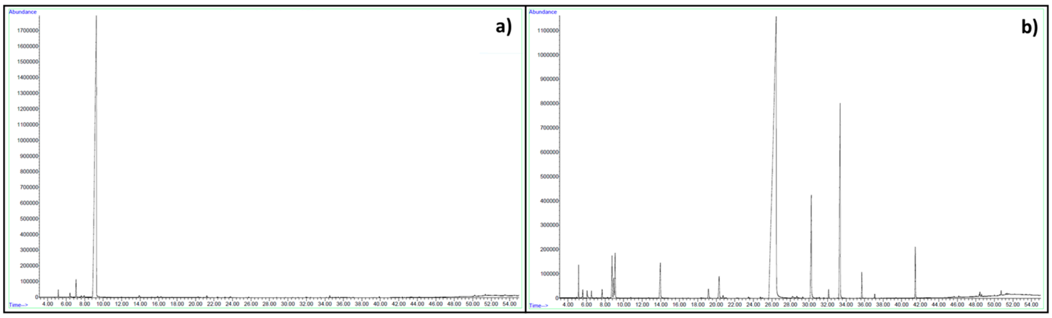

Figure S1. GC-MS chromatogram of EOs; (**a**) OEO and (**b**) CEO

3. DLS Analysis Results

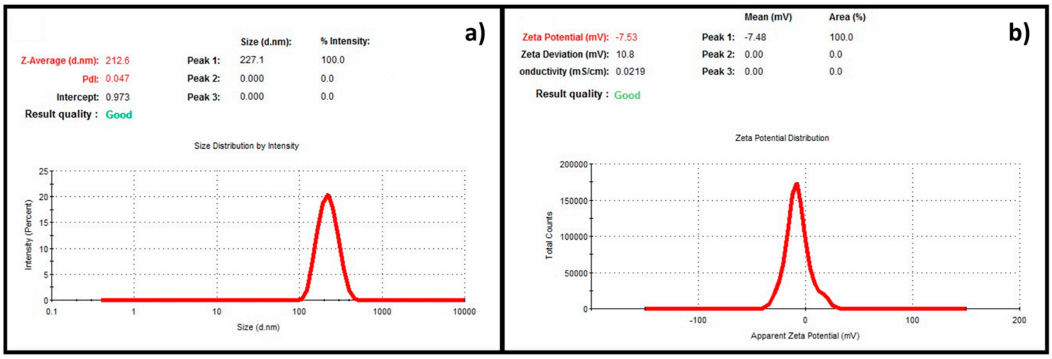

Figure S2. DLS analysis results: (a) average particle size, PdI and (b) zeta potential graphics of blank PLGA nanoparticles

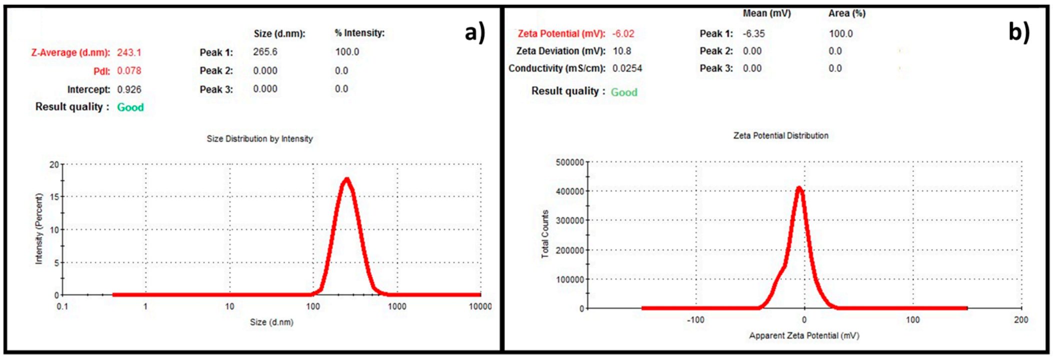

Figure S3. DLS analysis results: (**a**) average particle size, PdI and (**b**) zeta potential graphics of OEO loaded PLGA nanoparticles

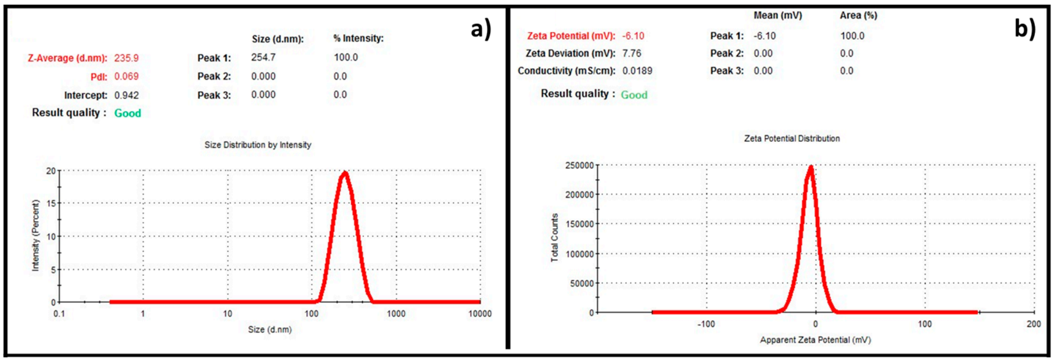

Figure S4. DLS analysis results: (**a**) average particle size, PdI and (**b**) zeta potential graphics of CEO loaded PLGA nanoparticles

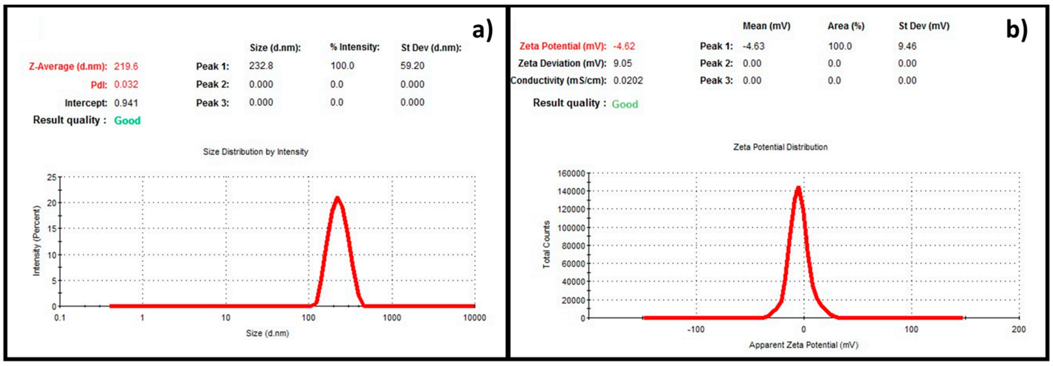

Figure S5. DLS analysis results: (**a**) average particle size, PdI and (**b**) zeta potential graphics of OEO-CEO loaded PLGA nanoparticles.
